# Toward Development of the Male Pill: A Decade of Potential Non-hormonal Contraceptive Targets

**DOI:** 10.3389/fcell.2020.00061

**Published:** 2020-02-26

**Authors:** Katarzyna Kent, Madelaine Johnston, Natasha Strump, Thomas X. Garcia

**Affiliations:** ^1^Department of Pathology & Immunology, Baylor College of Medicine, Houston, TX, United States; ^2^Department of Biology and Biotechnology, University of Houston–Clear Lake, Houston, TX, United States; ^3^Center for Drug Discovery, Baylor College of Medicine, Houston, TX, United States

**Keywords:** contraception, drug target, knockout mouse, spermatozoa, druggability

## Abstract

With the continued steep rise of the global human population, and the paucity of safe and practical contraceptive options available to men, the need for development of effective and reversible non-hormonal methods of male fertility control is widely recognized. Currently there are several contraceptive options available to men, however, none of the non-hormonal alternatives have been clinically approved. To advance progress in the development of a safe and reversible contraceptive for men, further identification of novel reproductive tract-specific druggable protein targets is required. Here we provide an overview of genes/proteins identified in the last decade as specific or highly expressed in the male reproductive tract, with deletion phenotypes leading to complete male infertility in mice. These phenotypes include arrest of spermatogenesis and/or spermiogenesis, abnormal spermiation, abnormal spermatid morphology, abnormal sperm motility, azoospermia, globozoospermia, asthenozoospermia, and/or teratozoospermia, which are all desirable outcomes for a novel male contraceptive. We also consider other associated deletion phenotypes that could impact the desirability of a potential contraceptive. We further discuss novel contraceptive targets underscoring promising leads with the objective of presenting data for potential druggability and whether collateral effects may exist from paralogs with close sequence similarity.

## Introduction

Currently, fertility control approaches for men fall into one of two categories: hormonal and non-hormonal. Several approaches have been investigated involving injectable or transdermal regimes using testosterone alone or combined with other molecules (Bagatell et al., 1993; Ilani et al., 2012). Although claims of total reversibility and full recovery to fertility have been made with hormonal contraception in males (Pasztor et al., 2017), prolonged use of exogenous hormones is associated with off-target effects, such as decreased high density lipoprotein cholesterol levels and potential cardiovascular risk in otherwise healthy men (Meriggiola et al., 1995). Therefore, there has been considerable interest in alternative methods for safe and reversible fertility control. Several non-hormonal methods are currently under development including gel-based obstruction of vas deferens, contraceptive vaccines, sperm-specific calcium ion channel blockers, and anti-spermatogenic indenopyridines with varied effectiveness and risks involved (Aitken, 2002; Hild et al., 2007; Morakinyo et al., 2009; Baggelaar et al., 2019).

## The ‘Popular’ Proteins: What Has Been Targeted So Far?

Past efforts on the development of a safe non-hormonal contraceptive for men has resided on a diverse array of targets identified through both forward and reverse approaches. Forward approaches include identification of a target protein prior to drug development, while reverse approaches identify a drug with contraceptive effect prior to identification of the target protein. Often reverse approaches fail to identify specific drug targets with minimal side effects as discussed below.

### Targets Involved in Sertoli-Germ Cell Interactions

Adjudin, a derivative of 1H-indazole-3-carboxylic acid, was shown to have potent anti-spermatogenic activity in rats, rabbits, and dogs prior to identification of its targets (Mok et al., 2011). Through further studies, it was later found to disrupt the Sertoli-germ cell junction proteins ACTB, ITGA6, ITGB1, MYH11, and OCLN (Mok et al., 2011, 2012; Mruk and Cheng, 2011). Unsurprisingly, administration of this drug resulted in significant toxicity, including effects on liver and skeletal muscle (Mruk et al., 2006) necessitating efforts to lower systemic toxicity by developing a conjugate capable of delivering Adjudin directly to the testis (Mruk et al., 2006), and by utilizing different formulations of the drug for its oral use to reduce the effective dose (Chen et al., 2016a). However, the efficacy of these approaches has yet to be determined.

H2-Gamendazole, an additional indazole carboxylic acid analog, blocks spermatogenesis by inhibiting production of inhibin B by primary Sertoli cells (Tash et al., 2008a), inhibiting HSP90AB1 and EEF1A1, and increasing Interleukin 1 Alpha expression in Sertoli cells (Tash et al., 2008b), which is a known disruptor of Sertoli cell-spermatid junction integrity (Nilsson et al., 1998). Although Gamendazole was shown to induce significant loss of spermatids (Tash et al., 2008b) and cause complete infertility after just a single dose, the damages to the seminiferous epithelium were not fully reversible (Tash et al., 2008a). The failure of some animals to regain fertility may have resulted from the combined effects of this drug on EEF1A1 and HSP90 in less differentiated spermatogenic cells. Therefore, further progress on this drug has been halted until these issues can be addressed. Generation of potentially safer analogs of gamendazole are currently under development (Kanishchev and Dolbier, 2018).

### Targets That Reside on the Testis Side of the Blood–Testis Barrier

One major obstacle in the development of a safe, reversible, and non-hormonal male contraceptive is identifying small molecule inhibitors that can penetrate the highly selective blood–testis barrier (BTB), or Sertoli cell barrier (SCB). Established through a strong network of tight junction proteins between adjacent Sertoli cells, the BTB/SCB partitions the seminiferous epithelium into two compartments: basal and adluminal (lumen-facing) (Franca et al., 2016). The basal compartment contains the undifferentiated spermatogonia, differentiating spermatogonia, and early differentiating spermatocytes. Meanwhile, the lumen-facing compartment contains differentiated spermatocytes, and round and elongated spermatids that—due to homologous recombination during meiosis—are no longer recognized by the immune system and thus require an immune privileged location. Many molecules have been shown to not cross the BTB/SCB and are thus used as molecular tools for measuring an intact BTB/SCB in manipulated animal models (Chen et al., 2018).

To the best of our knowledge, the small-molecule inhibitor JQ1, which inhibits the bromodomain-containing, testis-specific protein, BRDT, is currently the only molecule that has been shown to be present beyond the BTB/SCB (Matzuk et al., 2012). By binding to the BRDT acetyl-lysine binding pocket, JQ1 disrupts spermatogenesis at the spermatocyte and round spermatid stages, producing a reversible contraceptive effect in mice (Matzuk et al., 2012) that phenocopies the male infertility phenotype of BRDT knockout mice (Shang et al., 2007). Development of JQ1 was done in a target-based drug discovery approach focused on finding inhibitors of BRD4, a close paralog of BRDT. JQ1 blocks the production of sperm in the testes by inhibiting BRDT, however, it also acts on other bromodomains (BRD) of the bromo-and-extra-terminal (BET) proteins known to regulate transcription and DNA repair, which include important BRDT paralogs with oncogenic effects (French et al., 2008). BRD4, has become known as an important therapeutic target in several types of cancers, including breast and prostate cancers, as well as glioblastoma multiforme (GMB) (Shi et al., 2014; Zhou et al., 2016; Wen et al., 2019). JQ1 possesses anti-tumor effects (Shao et al., 2014; Das et al., 2015) and has been identified as a promising inhibitor for treating GBM (Wen et al., 2019). Along with other related pan-BET BRD compounds JQ1 was also associated with improvement of associated memory and enhancement of special memory precision in mice, showing potential for treatment of dementia (Benito et al., 2017). Given the therapeutic potential of JQ1 significant efforts have been made to understand the mechanisms of its action on the BET BRD family, giving insight into the intricate protein–protein interactions (PPI) with transcriptional complexes, and most importantly, providing means for enhancing inhibition specificity for a single BET (Lambert et al., 2019).

### Sperm Maturation in the Epididymis

The epididymis is a prime target for the development of a male contraceptive. This is because sperm leaving the testis are neither motile nor able to recognize or fertilize an egg; they must transit through the epididymis to acquire these abilities (Sullivan and Mieusset, 2016). Knockout mice of several epididymis-specific proteins have confirmed that the epididymis is essential for sperm maturation in the mouse (Davies et al., 2004; Roberts et al., 2006; Sipila et al., 2009). To the best of our knowledge, inhibitors that target an epididymis-specific protein do not yet exist. However, one of the most promising reversible, non-hormonal male contraceptives developed thus far has been against EPPIN (epididymal protease inhibitor; SPINLW1), which was developed through a reverse approach. EPPIN is a protein secreted by Sertoli cells and epithelial cells of the epididymis that gets deposited on the surface of maturing spermatozoa (Silva et al., 2012). The small molecule inhibitor EP055, which targets EPPIN, causes reversible infertility in primates (Silva et al., 2012) most likely by targeting the testes and epididymis where the drug can be found (O’Rand et al., 2018). In fact, deposition of EPPIN on the sperm surface appears to be greatest in the epididymis, indicating that the drug primarily acts in the epididymis (O’Rand et al., 2018). Current efforts are focused on increasing the half-life of the drug molecule (O’Rand et al., 2018).

Additionally, the inhibitors cyclosporine A and FK506, which are used as immunosuppressant drugs, target the sperm calcineurin subunits PPP3CC and PPP3R2 in the epididymis causing reversible effects on sperm morphology and motility (Miyata et al., 2015). Treatment of mice with cyclosporine A or FK506 creates phenocopies of the sperm motility and morphological defects apparent in knockout mice, which appear within 4 to 5 days of treatment and are reversed a week after discontinuation (Miyata et al., 2015). Unfortunately, cyclosporine A and FK506 are undesirable candidates due to their immunosuppressive effect (Stucker and Ackermann, 2011).

## Factors That Make a Promising Candidate Target

When evaluating potential druggability in a target-based drug discovery process, one must consider the protein properties that are required for safe and effective inhibition. Among the most significant is tissue expression specificity to minimize potential adverse effects; protein function and whether protein activity or interaction with other proteins is potentially druggable; sequence similarity to closely related paralogs that may be ubiquitously expressed; whether genetically manipulated animal models demonstrate a functional requirement for the target of interest; and other considerations as discussed below.

### Male Reproductive Tract Specificity

By ensuring in the first steps of development that candidate drug targets are near exclusively expressed in the male reproductive tract, the potential for off-target effects in humans is minimized. Gamendazole, for example, targets HSP90AB1 and EEF1A1, which are highly expressed in non-reproductive tissues (Djureinovic et al., 2014; Uhlen et al., 2015). Therefore, significant toxicity as evidenced through previous studies (Tash et al., 2008a, b) could have been predicted based on target gene expression analysis. Likewise, the toxicity of Adjudin has resulted in attempts to target the drug specifically to the testes through conjugation with FSH (Chen et al., 2016a), a complicated approach which may minimize, but not completely remove, off-target effects. Since the identified molecular targets of Adjudin are ACTB, ITGA6, ITGB1, MYH11, and OCLN (Mruk and Cheng, 2011; Mok et al., 2012), which are all widely expressed in non-reproductive organs (Djureinovic et al., 2014; Uhlen et al., 2015), likewise, the toxicity of Adjudin could have been predicted and avoided had the drug’s development began with a target-based discovery approach.

### Protein Druggability

Protein druggability is often based on the protein family for which other members are known drug targets (Hopkins and Groom, 2002). For instance, an enzyme with a known binding site might be an easier target when compared to a novel protein that has not already been categorized. The development of an inhibiting drug for an uncategorized protein might seem challenging, however, disrupting PPI has recently been gaining attention as one of the possible methods. Generally, PPI are considered to be more challenging than traditional drug targets due to the smaller protein interfaces and difficulty with finding a sufficiently binding ligand capable of interrupting the interaction site at a suitable concentration (Whitty and Kumaravel, 2006). However, PPI targets are not deemed undruggable, based on the discovery of small molecules capable of deeper and higher affinity binding within the contact surfaces of the target protein (Wells and McClendon, 2007). Therefore, although not initially compelling, uncategorized genes have the likelihood of becoming potential drug targets by using druglike compounds that can modulate the PPI at multiple interface sites—increasing the ligand binding affinity—and consequently lowering the necessary drug dose administered (Fuller et al., 2009). Table 1 presents the list of male reproductive tract-specific genes expressed at various stages of spermatogenesis with assigned categories of protein families. Besides a handful of enzymes (HFM1, MOV10L1, PGK2, PRSS37, and LRGUK), two transcription factors (SOX30 and TERB1), a few epigenetically active proteins (SCML2 and TDRD5) and a sperm specific ion channel KCNU1, most of the proteins discussed in this review belong to an unknown category. However, the contraceptive potential of these genes should not be overseen, but rather investigated for the identification of a high-affinity small molecule that can either (1) interfere with PPI, or (2) target the protein specifically for degradation as discussed below.

**TABLE 1:** Recently identified reproductive tract-specific genes with male infertility phenotypes in mice: potential non-hormonal male contraceptive drug targets.

Gene Symbol (Mouse/Human)	Drug Target Type	Spermatogenic Site of Expression	MW (kDa)					Reduced testis size in mouse	
								
			Mouse	Human	# TM	Secreted	Conditions underlying male infertility in mouse		References
*Asz1/ASZ1*	Unknown	Spermatogonia	53	53.5	0	No	increased hypomethylation of retrotransposons, germ cell loss due to apoptosis, Sertoli cell only phenotype	Yes	Ma et al., 2009
*Mov10l1/MOV10L1*	Enzyme	Spermatogonia	132.8	135.3	0	No	Abnormal spermatocyte morphology, arrest of male meiosis	Yes	Frost et al., 2010
*Scml2/SCML2*	Epigenetic	Spermatogonia	80.1	77.3	0	No	Abnormal spermiogenesis	Yes	Hasegawa et al., 2015
*Tex101/TEX101*	Unknown	Spermatogonia	21.1	26.7	0	No	Abnormal sperm physiology	No	Li et al., 2013
*4930447C04Rik/C14orf39*	Unknown	Spermatocytes	66.9	68.2	0	No	Azoospermia	Yes	Gomez et al., 2016
*Boll/BOLL*	Unknown	Spermatocytes	30.9	31.3	0	No	Azoospermia, abnormal male germ cell apoptosis, abnormal spermatogenesis	Yes	VanGompel and Xu, 2010
*Btbd18/BTBD18*	Unknown	Spermatocytes	79.5	77.9	0	No	Abnormal spermatid morphology, arrest of spermiogenesis	Yes	Zhou et al., 2017
*Ccdc155/CCDC155*	Unknown	Spermatocytes	72.4	62.8	0	No	Abnormal chromosomal synapsis, abnormal double-strand DNA break repair, abnormal meiotic attachment of telomere to nuclear envelope	Yes	Horn et al., 2013
*Ccdc63/CCDC63*	Unknown	Spermatocytes	65.2	66.3	0	No	Shortened sperm flagella	No	Young et al., 2015
*Cnbd2/CNBD2*	Unknown	Spermatocytes	77.9	67.5	0	No	Abnormal sperm midpiece morphology, abnormal sperm motility	Yes	Krahling et al., 2013
*Fbxo43/FBXO43*	Unknown	Spermatocytes	71.2	78.4	0	No	Arrest of male meiosis	Yes	Gopinathan et al., 2017
*Hfm1/HFM1*	Enzyme	Spermatocytes	161.4	162.6	0	No	Abnormal chiasmata formation, abnormal chromosomal synapsis, arrest of male meiosis	Yes	Guiraldelli et al., 2013
*Hormad2/HORMAD2*	Unknown	Spermatocytes	34.8	35.3	0	No	Abnormal chiasmata formation, arrest of spermatogenesis	Yes	Kogo et al., 2012a
*Insl6/INSL6*	Unknown	Spermatocytes	22.2	22.5	0	Yes	Azoospermia, abnormal male germ cell morphology	Yes	Burnicka-Turek et al., 2009
*Ly6k/LY6K*	Unknown	Spermatocytes	11.7	14.2	0	Yes	Abnormal sperm physiology	No	Fujihara et al., 2014
*Mcmdc2/MCMDC2*	Unknown	Spermatocytes	76	76.2	0	No	Abnormal double-strand DNA break repair, abnormal synaptonemal complex	Yes	Finsterbusch et al., 2016
*Meiob/MEIOB*	Unknown	Spermatocytes	53	49.3	0	No	Abnormal double-strand DNA break repair, abnormal meiosis, arrest of spermatogenesis	Yes	Luo et al., 2013; Souquet et al., 2013
*Meioc/MEIOC*	Unknown	Spermatocytes	108.9	107.6	0	No	Abnormal double-strand DNA break repair, abnormal synaptonemal complex	Yes	Abby et al., 2016
*Nup210l/NUP210L*	Unknown	Spermatocytes	205.1	206.6	1	Yes	Abnormal acrosome morphology, abnormal sperm midpiece morphology	Unknown	Walters et al., 2009
*Pgk2/PGK2*	Kinase	Spermatocytes	44.9	44.7	0	No	Asthenozoospermia	No	Danshina et al., 2010
*Pih1h3b/PIH1D3*	Unknown	Spermatocytes	24.5	24.1	0	No	Abnormal sperm axoneme morphology, abnormal sperm flagellum morphology	Yes	Dong et al., 2014
*Rad21l/RAD21L1*	Unknown	Spermatocytes	62.7	63.3	0	No	Azzospermia, abnormal male meiosis	Yes	Herran et al., 2011
*Sox30/SOX30*	TF	Spermatocytes	83.9	82.9	0	No	Abnormal spermatid morphology, arrest of spermiogenesis	Yes	Feng et al., 2017
*Spata22/SPATA22*	Unknown	Spermatocytes	40.2	41.3	0	No	Abnormal chromosomal synapsis, abnormal double-strand DNA break repair, abnormal synaptonemal complex	Yes	La Salle et al., 2012
*Spdya/SPDYA*	Unknown	Spermatocytes	36.1	36.5	0	No	Abnormal chiasmata formation, abnormal chromosomal synapsis, abnormal synaptonemal complex	Yes	Tu et al., 2017
*Syce3/SYCE3*	Unknown	Spermatocytes	10.5	10.6	0	No	Abnormal chromosomal synapsis, abnormal synaptonemal complex	Yes	Schramm et al., 2011
*Tdrd5/TDRD5*	Epigenetic	Spermatocytes	116	109.7	0	No	Globozoospermia, abnormal acrosome morphology, abnormal spermatocyte morphology, arrest of male meiosis	Yes	Yabuta et al., 2011
*Terb1/TERB1*	TF	Spermatocytes	86.8	83.1	0	No	Abnormal X-Y chromosome synapsis during male meiosis	Yes	Shibuya et al., 2014
*Topaz1/TOPAZ1*	Unknown	Spermatocytes	185.5	191	0	No	Abnormal male germ cell morphology, arrest of male meiosis	Yes	Luangpraseuth-Prosper et al., 2015
*Calr3/CALR3*	Unknown	Spermatids	42.2	42.9	0	Yes	Abnormal sperm physiology	No	Ikawa et al., 2011
*Ccdc42/CCDC42*	Unknown	Spermatids	38	38	0	No	Abnormal sperm axoneme morphology, abnormal sperm head morphology, absent sperm flagellum, detached acrosome	No	Pasek et al., 2016
*Ccdc62/CCDC62*	Unknown	Spermatids	79.3	77.7	0	No	Abnormal sperm head morphology, abnormal sperm midpiece morphology, abnormal sperm motility, absent acrosome	No	Li et al., 2017
*Lrguk/LRGUK*	Enzyme	Spermatids	93.2	124.9	1	No	Oligoasthenoteratospermia, manchette dysfunction, abnormal sperm head shaping	Yes	Liu et al., 2015
*Prss37/PRSS37*	Enzyme	Spermatids	24.7	24.3	0	Yes	Abnormal sperm physiology, abnormal spermiogenesis, impaired fertilization	Yes	Shen et al., 2013
*Rimbp3/RIMBP3B*	Unknown	Spermatids	177.3	181	0	No	Detached acrosome, detached sperm flagellum, ectopic manchette	No	Zhou et al., 2009
*Spaca1/SPACA1*	Unknown	Spermatids	30.6	29.3	1	Yes	Abnormal acrosome morphology, abnormal sperm head morphology, coiled sperm flagellum	No	Fujihara et al., 2012
*Tcte1/TCTE1*	Unknown	Spermatids	55.5	55.6	1	No	Axoneme dysfunction, abnormal sperm motility	No	Castaneda et al., 2017
*Tdrd12/TDRD12*	Epigenetic	Spermatids	137.6	132.6	0	No	Abnormal spermatid morphology, arrest of male meiosis	Yes	Asano et al., 2009; Pandey et al., 2013
*Atp1a4/ATP1A4*	Transporter	Spermatozoa	114.9	114.2	10	No	Impaired sperm capacitation, kinked sperm flagellum	No	Jimenez et al., 2011
*Catsperd/CATSPERD*	Unknown	Spermatozoa	89.5	90.4	1	Yes	Abnormal germ cell morphology, reduced hyperactivated sperm motility	No	Chung et al., 2011
*Cfap54/CFAP54*	Unknown	Spermatozoa	353.8	352	0	No	Abnormal sperm axoneme morphology, absent sperm flagellum, short sperm flagellum	No	McKenzie et al., 2015
*Kcnu1/KCNU1*	IC	Spermatozoa	126.9	129.5	7	No	Impaired acrosome reaction, impaired fertilization, impaired sperm capacitation	No	Santi et al., 2010; Zeng et al., 2011
*Pmis2/PMIS2*	Unknown	Spermatozoa	11	15.8	2	No	Abnormal sperm physiology	Unknown	Yamaguchi et al., 2012
*Rsph6a/RSPH6A*	Unknown	Spermatozoa	80.2	80.9	0	No	Abnormal manchette morphology, abnormal sperm axoneme morphology, abnormal sperm fibrous sheath morphology	No	Abbasi et al., 2018
*Sun5/SUN5*	Unknown	Spermatozoa	42.7	43.1	2	No	Globozoospermia, sperm head detachment	No	Shang et al., 2017

*TM, transmembrane.*

Many of the potential drug targets, such as non-enzymatic proteins, are uncategorized and identified as “undruggable” due to various challenges with existing targeting approaches. However, an emerging targeted protein degradation method called Proteolysis Targeting Chimeras (PROTACs) is a promising technique that can address these issues. The traditional approach to most enzymatic proteins serves to interfere with the functional aspect of the protein target, whereas PROTACs eliminate the protein through utilization of the ubiquitin-proteasome system to promote selective degradation (Bondeson et al., 2015; Lu et al., 2015; Olson et al., 2018). Thus, PROTACs do not require that an identified small molecule both bind with high affinity and reduce activity of the target protein (through interference of the binding pocket, etc.). PROTACs only requires that the identified small molecule bind with high affinity to the protein target. Additional design and chemistry are conducted to conjugate the small molecule to a high affinity E3 ubiquitin ligase ligand that results in target protein ubiquitination. There are currently various combinations of PROTACs developed to overcome the limitations of cell permeability, stability, solubility, selectivity, and tissue distribution (Brooks et al., 2005; Bechara and Sagan, 2013; Ottis et al., 2017; Bondeson et al., 2018). Therefore, PROTACs technology provides the potential to greatly promote the development of contraceptive drugs against the “undruggable” non-enzymatic protein targets.

### Sequence Similarity to Known Paralogs

The probability of two or more paralogs sharing a specific function increases with the percentage of sequence similarity (Zallot et al., 2016). If a reproductive tract-specific target is found to have high sequence similarity to a ubiquitously expressed protein or paralog, especially in the potentially druggable domain, this would make the target a poor choice for contraceptive development due to potential off-target effects. However, if a reproductive tract-specific protein has one or more ubiquitously expressed paralogs with low sequence similarity, this indicates that the function of the expressed proteins in the reproductive tract could be disrupted without significant off-target risks. Considerably high contraceptive potential resides in genes without a confirmed paralog; however, one must remain cautious in assuming a complete lack of paralogs as the druggable domain may be present in completely unrelated proteins, and further investigation should be conducted before proceeding to contraceptive drug development.

### Validation Through Ablation – Creating Functional Knockouts to Verify Contraceptive Potential

Mice serve as one of the most efficient and effective models to understanding human physiology for a variety of reasons. The mouse genome is very well-characterized with almost all genes sharing similar functions to human orthologs (Cheng et al., 2014; Lin et al., 2014). Mice are biologically very similar, yet because they are small and have very short lifespans compared to humans, developmental processes can be studied economically and at an accelerated rate. Genetically manipulated knockout mouse models have significantly advanced our understanding of male gamete differentiation and the molecular mechanisms underlying male fertility. Nevertheless, there is still much to be learned about these intricate processes considering only approximately half of the protein coding genes have been individually ablated in mice (Dickinson et al., 2016; Smith et al., 2018). By ensuring that a knockout animal model of an individual protein target of interest leads to a complete male infertility phenotype, not subfertility, confidence in potential drug efficacy is established.

The current popularity of CRISPR-generated knockout mouse models as a method to study the function of a gene *in vivo* has dominated over other approaches. While knockout methods lead to a complete depletion of gene expression, or complete elimination of gene function, methods involving gene knockdowns lead to reduction in protein expression, the results of which may yield valuable information. RNA interference (RNAi) was found to be useful for silencing gene expression in mammals and employed to study the functional relevance of genes in a relatively fast and easy way. Past reproductive studies have utilized short interfering RNA (siRNA) to determine functional significance of genes in male mouse fertility and has, in some cases, resulted in impaired or complete infertility phenotype in viable mice (Nagai et al., 2011; Welborn et al., 2015). Several interesting experiments that compared the phenotypes resulting from knocking-out and knocking-down mouse genes have revealed that the two methods are complimentary in conducting gene functional studies (De Souza et al., 2006). The focus of this review, however, lies in male reproductive genes whose functional relevance was determined through knockout mouse models, since incisive genetic approaches provide a more definitive foundation for establishing the contraceptive potential of a novel reproductive tract-specific gene. A variable and indeterminate level of off-target effects introduced from transfection or lentivirus-mediated infection cannot be excluded as confounding variables in knockdown experiments.

Many reproductive tract-specific genes have been identified as dispensable for male fertility when individually ablated in mice (Miyata et al., 2016; Holcomb et al., 2020; Lu et al., 2019). Functional compensation through upregulation of other functionally similar genes or molecular pathways may explain the lack of phenotype (Marschang et al., 2004; Hanada et al., 2009; Kashiwabara et al., 2016). Therefore, identifying a small molecule inhibitor against an individually dispensable target would yield no contraceptive effect.

As previously discussed, BRDT is a testis-specific gene that when individually ablated in mice results in arrest of male meiosis, azoospermia, and complete male infertility (Barda et al., 2016). Since BRDT has enough sequence dissimilarity—>55% sequence dissimilarity with its closest paralogs that are ubiquitously expressed, BRD4 and BRD2, development of BRDT inhibitors with enhanced specificity and drug selectivity is feasible and currently underway (Matzuk et al., 2012; Miller et al., 2016). On the other end of the spectrum, GJA1 is an example of a gene that when conditionally ablated in the Sertoli cells of male mice leads to male infertility (Brehm et al., 2007). However, because the gene is not exclusively expressed in the reproductive tract and (Djureinovic et al., 2014; Uhlen et al., 2015) other knockout animal models of this gene display a variety of non-reproductive phenotypes including severe heart abnormalities (Gutstein et al., 2001; Liao et al., 2001), thus demonstrating that this target is not suitable for the development of a safe male contraceptive.

### Human Mutations Implicated in Male Infertility

**FIGURE 1:**
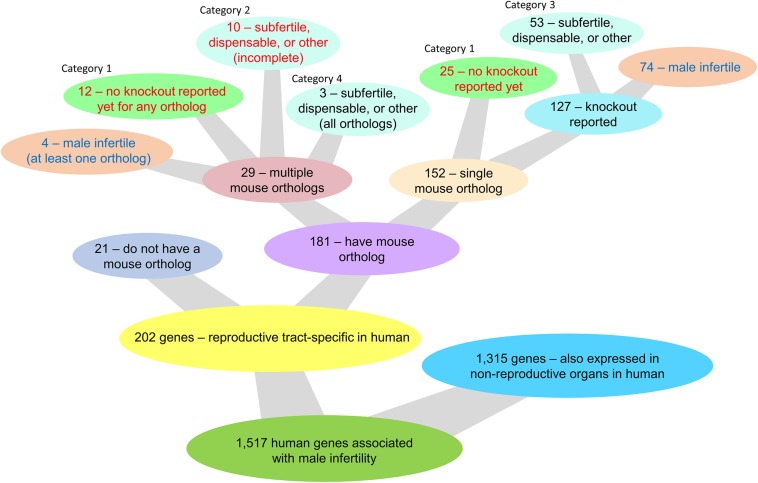
Knockout mouse model availability and phenotype outcome of single and multiple mouse ortholog genes corresponding to 202 reproductive tract-specific human genes associated with male infertility and/or either azoospermia, globozospermia, or oligospermia as reported by GeneCards and MalaCards (Stelzer et al., 2016; Rappaport et al., 2017).

**FIGURE 2:**
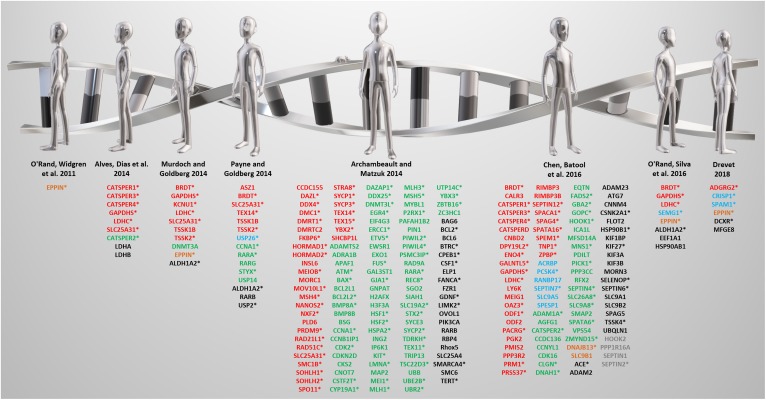
A review of reviews from the past decade. Targets identified by the listed reviews are color-coded based on reproductive tract-specificity and fertility phenotype as follows: **red** = reproductive tract-specific displaying male infertility phenotype; **green** = non-reproductive tract-expressed and male infertility phenotype; **blue** = reproductive tract-specific displaying fertile or subfertile phenotype; **orange** = reproductive tract-specific with unknown fertility phenotype; **black** = non-reproductive tract-expressed displaying fertile or subfertile phenotype; **gray** = non-reproductive tract-expressed with unknown fertility phenotype. Expression and phenotype data obtained from Contraceptive Infertility Target DataBase (CITDBase), Human Protein Atlas (HPA), Ensembl Biomart, Mouse Genome Informatics (MGI), and the International Mouse Phenotyping Consortium (IMPC). *denotes genes implicated in human male infertility according to GeneCards and MalaCards.

Mutations causing male infertility in humans might also be informative in the context of contraception and should be taken into consideration in the search for a potential non-hormonal target. There are 1,517 human genes associated with male infertility and/or either azoospermia, globozospermia, or oligospermia as reported by GeneCards and MalaCards (Stelzer et al., 2016; Rappaport et al., 2017). Of these 1,517 genes, 202 genes are reproductive tract-specific in humans as reported through the CITDBase Contraceptive Target Database (GTEx Consortium, 2013; Djureinovic et al., 2014; Uhlen et al., 2015; Schmidt et al., 2017; Lee, 2019; Samaras et al., 2019) and/or Djureinovic et al. (2014) and Uhlen et al. (2015) (Figure 1). Of these 202 genes, 21 genes do not have a corresponding mouse ortholog and 3 of these genes (*KLK2*, *CDY2A*, and *RHOXF2*) may be of potential interest for further study as they encode an enzyme, an epigenetic protein, and a transcription factor, respectively; all proteins with potential druggable activity (Supplementary Table S1). The remaining 18 genes without a corresponding mouse ortholog encode proteins of unknown drug target type (Supplementary Table S1). Of the 202 reproductive tract-specific human genes associated with human infertility, 78 genes have a corresponding mouse ortholog displaying male infertility in the mouse (Figure 1 and Supplementary Table S1). The remaining 124 genes either (1) have a single mouse ortholog each and none have been knocked-out in the mouse, (2) have two or more mouse ortholog genes, whereby none or an incomplete number of orthologs have been knocked-out, and of those that have been knocked-out, none display a male infertility phenotype, (3) have a single mouse ortholog or multiple mouse orthologs that when individually ablated all lack a male infertility phenotype, or (4) have multiple mouse orthologs that have all been knocked out and that individually do not display a male infertility phenotype (Figure 1 and Supplementary Table S1). While the first two categories (categories 1 and 2) require further study for functional validation of the contraceptive potential of these genes, the latter categories (categories 3 and 4) do not require any further study. Genes listed in the first two categories (47 genes total) encode 2 enzymes (*KLK3* and *SPAM1*), 1 kinase (*TSSK2*), 3 transcription factors (*HSFY1*, *TGIF2LX*, and *TGIF2LY*), and 41 proteins of unknown drug target type (Supplementary Table S1). Of the 202 reproductive tract-specific human genes associated with human infertility, 32 genes are mentioned in Figure 2, and four genes (*BOLL*, *KCNU*, *SPATA22*, and *TEX101*) are discussed in further detail within this review.

### Site of Target Expression

Potential contraceptives could hit targets expressed along various stages of sperm development in the testis and epididymis, including targets active during the maintenance of the progenitor spermatogonia pool, entry into and passage through the various stages of meiosis, spermatid development and release, and sperm maturation through the epididymis. The contraceptive potential at these various stages varies in advantages and disadvantages. Targeting genes involved in early sperm development, for instance, could potentially be more effective, as suggested by several previous reviews identifying groups of male reproductive tract-expressed genes as promising drug targets (Schultz et al., 2003; Archambeault and Matzuk, 2014; Payne and Goldberg, 2014; O’Rand et al., 2016). However, disrupting the early stages of spermatogenesis poses the risk of testicular atrophy, longer recovery, and an increased possibility of irreversibility. Genes involved in later phases of spermatogenesis, namely spermiogenesis, acrosome and flagella formation, spermiation, and sperm maturation, would be more desirable contraceptive targets because testicular size would most likely remain unaffected, with a quicker, more reliable return to full fertility. Functional analysis of the expression patterns and specificity of male reproductive tract genes is imperative as it often provides additional insight into the molecular mechanisms of various stages of spermatogenesis, understanding of which is necessary in the process of developing a safe and effective non-hormonal male contraceptive.

### Other Considerations

Identification of a drug-like small molecule that can effectively modulate the activity of a given target should also be assessed based on protein properties such as structure, size, and complexity (Kozakov et al., 2015). Targets that contain transmembrane helices could increase the difficulty of obtaining a properly folded and soluble protein for drug selection purposes. Likewise, due to glycosylation and processing through the secretory pathway, secreted proteins require protein production in eukaryotic expression systems, which could increase the difficulty of obtaining suitable quantities of purified protein. Therefore, whether drug development is feasible resides on careful consideration of the biophysical properties of the protein.

## Target Genes

### Previously Reviewed ‘Novel’ Target Genes

Several notable reviews published in the last decade have mentioned promising, non-hormonal, contraceptive leads that include both meiotically and post-meiotically expressed genes that are testis-specific or epididymis-specific genes required for sperm maturation (O’Rand et al., 2011; Alves et al., 2014; Archambeault and Matzuk, 2014; Murdoch and Goldberg, 2014; Payne and Goldberg, 2014; Chen et al., 2016b; O’Rand et al., 2016; Drevet, 2018) (Figure 2). Some are, in fact, not reproductive tract-specific, but are still required for fertility, while others that are indeed reproductive tract-specific, lead to subfertility, not infertility, which is an ineffective and highly undesirable outcome for a contraceptive. Thus, the genes in Figure 2 are color-coded according to reproductive tract-specificity and infertility phenotype in the mouse. It is worth noting that while the reviews were restricted to the past decade, the reproductive tract-specific genes mentioned in these reviews were first identified beyond the past decade including some that were reported in 2006 and earlier, such as TNP1 (Yu et al., 2000), CATSPER1 (Ren et al., 2001), and TEX14 (Greenbaum et al., 2006).

### In This Review

**FIGURE 3:**
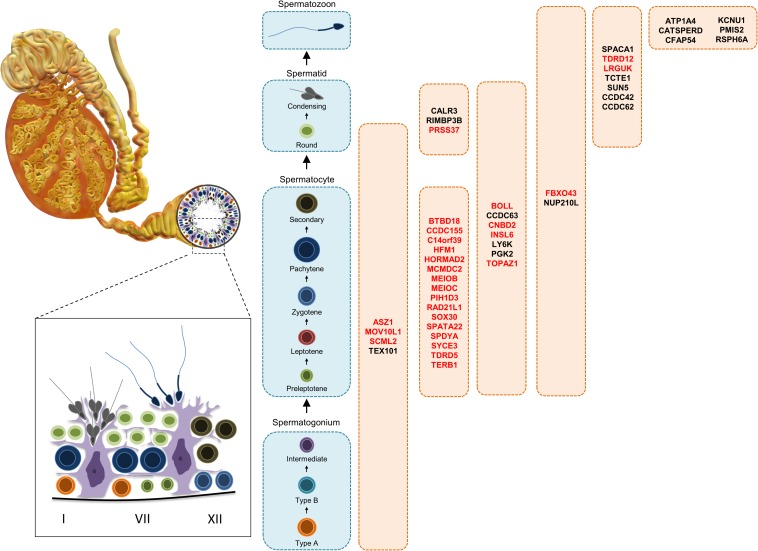
Diagrammatic representation of germ cells along the spermatogenic pathway showing the cell types displaying expression of the potential targets. Targets in red display decreased testes size in mice.

**FIGURE 4:**
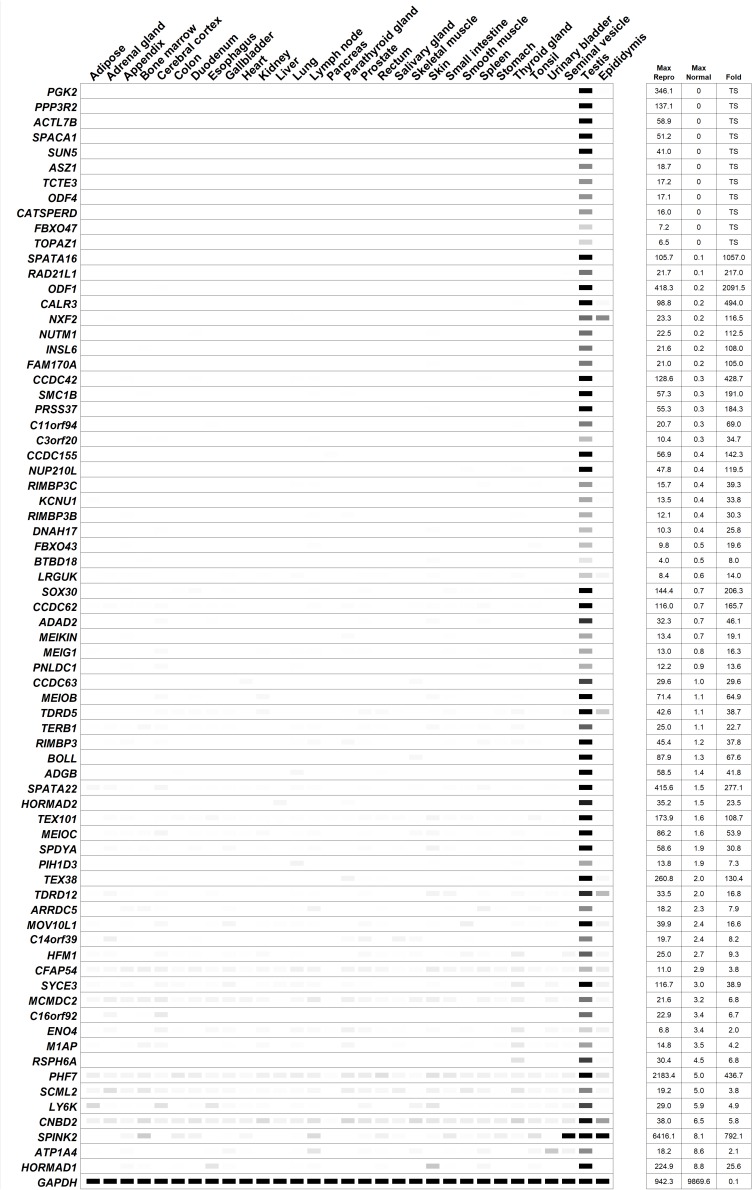
Digital PCR (heatmap) depicting the average transcripts per million (TPM) value per tissue per gene from human RNAseq data published by the Human Protein Atlas (Djureinovic et al., 2014; Uhlen et al., 2015). White = 0 TPM, Black ≥ 30 TPM. The genes are ordered from most reproductive tract-specific to least based on the level of non-reproductive tissue expression. “Fold” = testis/max normal. The data was obtained from a tab-separated file including Ensembl gene identifier, analyzed sample, and TPM value per gene that was downloaded from the Human Protein Atlas website. The expression profile of the housekeeping genes, *GAPDH*, is included.

In this review, 45 genes are discussed in further detail as they were identified in the last decade as required for male fertility through knockout mouse studies, whereby many of these genes were not discussed or discussed minimally in any previous male contraceptive drug target review. Henceforth, these 45 genes will be listed according to their published expression patterns (Figure 3), which is an important criterium that should be considered in the selection of a safe and/or desirable contraceptive target. It is worth noting that nearly all of the genes mentioned below are male reproductive tract-specific or highly enriched as reported in the literature and with additional confirmation through the published expression data from CITDBase (GTEx Consortium, 2013; Uhlen et al., 2015; Schmidt et al., 2017; Lee, 2019; Samaras et al., 2019) and/or the Human Protein Atlas (Djureinovic et al., 2014; Uhlen et al., 2015). A graphical summary of the RNAseq-based expression data for these 45 genes is depicted in Figure 4. Those that are not reproductive tract-specific are identified as such through the level of non-reproductive tissue expression. The following discussion provides detailed information about individual genes with focus on determining the contraceptive potential of each. Additional relevant information for these genes is listed in Table 1.

### Spermatogonia

In the last decade, only four reproductive tract-specific genes with infertile mouse models have been identified that fit the criteria of being expressed as early as the spermatogonia stage: *ASZ1* (Ma et al., 2009), *MOV10L1* (Frost et al., 2010; Zheng et al., 2010), *SCML2* (Hasegawa et al., 2015), and *TEX101* (Fujihara et al., 2013; Li et al., 2013) (Table 1). With expression as early as the spermatogonia stage (Teng et al., 2006; Ma et al., 2009; Frost et al., 2010; Maezawa et al., 2018), a deficiency of these genes’ functions will likely require at least 2 months prior to a contraceptive effect, at least 2 months for recovery, and in some cases, depending on the gene, the potential for irreversibility of the contraceptive effect. Nevertheless, since this form of contraceptive may be considered desirable due to the permanence of the effect, and the need for a compound that can traverse the BTB is not required, these genes are worthy of consideration. Since the conserved domains of human ASZ1, MOV10L1, and TEX101 show sequence similarity below 40% to their respective, non-reproductive tract expressed paralogs, ANKRD34C, CT55, and CD177, then these protein targets have a reasonable potential for drug specificity with a low risk of collateral effects. However, as it has been previously reported that human SCML2 is expressed ubiquitously at low levels (Bonasio et al., 2014) (Figure 4), low but potentially physiologically relevant expression of *SCML2* across many non-reproductive tissues may therefore be of concern in the process of drug development. Additionally, the closest paralog to SCML2, SCMH1, is ubiquitously expressed and shows 60% sequence similarity in its conserved domain. Based on this information, human SCML2 may not be an ideal contraceptive target.

### Spermatocytes

The genes discussed here are expressed in spermatocytes during preleptotene, leptotene, zygotene, pachytene, and/or diplotene stages and typically serve an essential role in meiosis I or meiosis II. A deficiency of these early stage genes results in male infertility typically due to meiotic arrest, which indicates a strong contraceptive potential. Important to note: although these genes may not directly impact spermatogonial stem cell self-renewal, and a healthy pool of spermatogonial stem cells may still remain for the regeneration of spermatogenesis, recovery time following cessation of targeting these genes may still take considerably longer than drugs targeting later stages of spermatogenesis or sperm. Consistently, the onset of a reliable contraceptive effect could take weeks, which may be an undesirable timeframe for drug action.

In the last decade, at least twenty-five reproductive tract-specific genes with infertile mouse models have been identified that fit the criteria of being expressed in spermatocytes (Table 1). Of these, seven genes—*MEIOB*, *MEIOC*, *PIH1D3*, *SPATA22*, *SYCE3*, *TERB1*, and *TOPAZ1*—encode the most promising target candidates as they do not have an associated paralog, or conserved domain with sequence similarity to any known protein, and thus these proteins bear the lowest risks of generating off-target effects from inhibiting compounds. Additionally, HORMAD2 is also an attractive target considering it only has one paralog, HORMAD1, which is also highly enriched in the male reproductive tract and leads to male infertility phenotype when ablated in mice (Shin et al., 2010; Daniel et al., 2011; Kogo et al., 2012b). Although *HORMAD1* shows low, but appreciable gene expression in two non-reproductive tissues (Figure 4) this fact may be offset by the sequence dissimilarity between HORMAD1 and HORMAD2 (50% whole protein and 40% conserved domain) which can aid in the identification of a selective inhibitor with careful drug selection and design. C14orf39 and CNBD2 would both be attractive candidates because they also lack paralogs, however both have tissue specificity issues of concern (Figure 4), and *Cnbd2* null mice display incomplete penetrance (Krahling et al., 2013), which may translate to potential ineffectiveness in humans. Therefore, while MEIOB, MEIOC, PIH1D3, SPATA22, SYCE3, TERB1, and TOPAZ1 are ideal candidates, C14orf39 and CNBD2 are not.

The next most promising candidates (another six genes)—*BTBD18*, *CCDC63*, *CCDC155*, *HFM1*, *MCMDC2*, and *TDRD5*—encode proteins that show sequence similarity below 40% to their respective, non-reproductive tract expressed paralogs, and within the conserved domains of their paralogs, KLHL26, SSH2, CCDC114, SNRNP200, MCM3, and TDRD1; thus, these potential targets have a reasonable potential for drug specificity with a low risk of collateral effects. However, the following six candidates—BOLL, FBXO43, INSL6, NUP210L, SOX30, and SPDYA—may require careful drug selection and design since the conserved domains of these proteins are 48–60% similar to the conserved domains of their respective, non-reproductive tract expressed paralogs, DAZ4, FBXO5, RLN1, NUP210, SOX7, and SPDYC. With respect to NUP210L, spermatid-Sertoli cell interaction was severely impaired in the knockout mouse model, resulting in Sertoli cell degeneration (Walters et al., 2009). Since this may cause irreversible disruption of spermatogenesis, this candidate may pose potential irreversibility issues. LY6K would be an attractive candidate because its closest ubiquitously expressed paralog, GML, has only 28% sequence similarity to LY6K; however, low, but appreciable expression of *LY6K* in non-reproductive tissues is of concern (Figure 4).

The most difficult spermatocyte-expressed candidates to target specifically are RAD21L1 and PGK2, which have ubiquitously expressed paralogs, RAD21 and PGK1, that have 83 and 87% similarity in their respective conserved domains. Since human mutations in RAD21 and PGK1 are characterized by significant disorders affecting numerous organ systems—Cornelia de Lange syndrome 4 (Deardorff et al., 2012), Mungan syndrome (Bonora et al., 2015), and phosphoglycerate kinase 1 deficiency (Fermo et al., 2012)—extraordinary effort would need to be made to generate specific drug molecules against RAD21L1 and PGK2.

### Round and Elongated Spermatids

The proteins discussed here are expressed during the later stages of spermatogenesis and sperm maturation (Table 1). These proteins are found in round or elongating spermatids, either in the acrosome, acroplaxome, basal body, manchette, or flagellum during spermiogenesis, and these proteins function in either proper sperm head formation and attachment, midpiece formation, acrosome formation and attachment, generating sperm with normal motility, or generating sperm capable of normal sperm-zona binding. Targeting genes at these post-meiotic stages is more likely to act within a faster timeframe and lead to better and potentially faster recovery of fertility upon cessation of drug.

In the last decade, at least nine reproductive tract-specific genes with infertile mouse models have been identified that fit the criteria of being expressed in spermatids (Table 1). Again, the most attractive candidates are those that show excellent reproductive tract-specificity and that do not have any ubiquitously expressed paralogs, or that have ubiquitously expressed paralogs with low sequence similarity. CCDC62, SPACA1, and TCTE1 do not have any paralogs and TDRD12, CCDC42, PRSS37, and LRGUK have ubiquitously expressed paralogs, TDRD15, CFAP73, KLK15, and PPP1R42, that share less than 40% sequence similarity at the whole protein and conserved domain level. CALR3 has a ubiquitously expressed paralog, CALR, that shows 51% and 55% sequence similarity at the whole protein and conserved domain level, respectively, which may necessitate careful drug selection and design to ensure off-target effects are minimized. RIMBP3, RIMBP3B, and RIMBP3C in humans are all testis-specific and RIMBP3B and RIMBP3C share conserved domains with identical protein sequences, indicating a strong evolutionary requirement for the function of these proteins and high likelihood of targeting more than one isoform with one small molecule inhibitor. However, the closest ubiquitously expressed paralog to these proteins is RIMBP2, which shares 76% sequence similarity in its conserved domain, which may be of concern in finding a specific drug molecule that targets this region.

### Spermatozoa

Genes in this group are highly expressed in spermatozoa and disrupting their function would most likely affect spermatozoa maturation as they pass through the epididymis, while still maintaining the pool of testicular spermatogonia and spermatocytes, or inhibit proper spermatozoa function after sperm maturation. These targets are typically localized to the neck, principal piece, flagellum, or the central microtubule apparatus of mature spermatozoa. Since these targets act late, it is important to note that some—depending on the binding kinetics of the drugs and if the drugs are reversible or irreversible inhibitors—may only provide a momentary decrease in sperm function while in the male reproductive tract, and shortly thereafter, but not indefinitely in the female reproductive tract after ejaculation as the drug concentration invariably decreases over time. Thus, although this category of drug may be the most desirable due to having fastest onset of drug action, fastest recovery after cessation of drug, and no effect on testicular size, there may be additional challenges to address during drug development to ensure contraceptive efficacy.

In the last decade, at least seven reproductive tract-specific genes with infertile mouse models have been identified that fit the criteria of being present in mature sperm (Table 1). CATSPERD, KCNU1, PMIS2, and SUN5 show the lowest potentials for off-target effects as their closest ubiquitously expressed paralogs, CASC4, KCNMA1, SYNDIG1L, and SUN1, have less than 50% similarity at the whole protein and conserved domain level. After that, RSPH6A, has 52% (whole) and 63% (CD) similarity to its closest ubiquitously expressed paralog, RSPH4A, which may make it a promising lead with additional attention during drug selection and design to minimize off-target effects. Although, CFAP54 would be an excellent candidate with less than 22% similarity (whole and CD) to its closest ubiquitously expressed paralog, NPBWR2, expression in cilia of the respiratory epithelium (Djureinovic et al., 2014; Uhlen et al., 2015) and additional low but potentially physiologically relevant expression across many non-reproductive tissues (Figure 4) indicate targeting this candidate may yield side effects. Likewise, ATP1A4 is not an ideal candidate due to having high sequence similarity—81% similarity (whole and CD)—to its closest ubiquitously expressed paralog, ATP1A2, and also having low but potentially physiologically relevant expression levels across many non-reproductive tissues (Figure 4).

### Additional Potential Targets

Not discussed in terms of potential drug target specificity, however of additional potential interest are the following genes that are also reproductive tract-specific in humans according to CITDBase (GTEx Consortium, 2013; Uhlen et al., 2015; Schmidt et al., 2017; Lee, 2019; Samaras et al., 2019) with mouse models displaying male infertility phenotype published in peer-reviewed journals in the last 10 years: 3 genes encoding enzymes [*ENO4* (Nakamura et al., 2013), *PNLDC1* (Nishimura et al., 2018), and *SPINK2* (Lee et al., 2011)] and 8 genes encoding proteins of unknown drug target type [*M1AP* (Arango et al., 2013), *MEIG1* (Zhang et al., 2009), *MEIKIN* (Kim et al., 2015), *NXF2* (Pan et al., 2009), *ODF1* (Yang et al., 2012), *PPP3R2* (Miyata et al., 2015), *SMC1B* (Revenkova et al., 2010), and *SPATA16* (Fujihara et al., 2017)]. Furthermore, the following genes are reproductive tract-specific in humans according to CITDBase (GTEx Consortium, 2013; Uhlen et al., 2015; Schmidt et al., 2017; Lee, 2019; Samaras et al., 2019) with mouse models displaying male infertility phenotypes as reported by the International Mouse Phenotyping Consortium (IMPC) (Munoz-Fuentes et al., 2018): 1 gene encoding an enzyme (*ADAD2*); 1 gene encoding an epigenetic-related protein (*PHF7*); and 11 genes encoding proteins of unknown drug target type (*ACTL7B*, *ADGB*, *ARRDC5*, *C11orf94*, *C16orf92*, *C3orf20*, *DNAH17*, *FBXO47*, *NUTM1*, *ODF4*, *TEX38*). The expression pattern of these additional individually published and IMPC-reported genes are listed in Figure 4.

## Conclusion

Global demand for the development of a “male pill” is at an all-time high with the exponential growth of the human population. Efforts to reduce unplanned pregnancies are recognized as the search for a safe and effective method of male contraception has been a decades-long quest (Prasad and Rajalakshmi, 1976; Frick and Aulitzky, 1988; Herndon, 1992; Waites, 1993; Gottwald et al., 2006; Mruk, 2008). Identification of reproductive tract-specific targets through transcriptomic and proteomic approaches followed by validation of their functional requirement using knockout mice models has helped advance this quest. In this review, we discuss novel reproductive tract-specific protein targets that have been identified in the past 10 years, their potential druggability, the factors that contribute to their druggability and why they should be taken into consideration when selecting male contraceptive targets. We direct the reader to consider that optimal gene targets are those that contribute to the later phases of spermatogenesis as disrupting genes in the earlier phases could potentially lead to permanent infertility. Additionally, the reader is directed to consider the potential adverse effects that may exist when targets whose protein sequences bear high sequence similarity to other ubiquitously expressed proteins. This information can be valuable in future studies since off-target effects can considerably hamper development of a safe, non-hormonal male contraceptive. Continued persistence in the search for an optimal protein target should lead to a clinically approved, affordable product.

## Author Contributions

KK and TG designed the research and wrote the manuscript. KK, MJ, NS, and TG performed the research and analyzed the data.

## Conflict of Interest

The authors declare that the research was conducted in the absence of any commercial or financial relationships that could be construed as a potential conflict of interest.

## Supplementary Material

TABLE S1 Orthologous mouse genes and mouse model information of 202 reproductive tract-specific human genes associated with male infertility in humans. Mouse ortholog gene symbols and phenotype information collected from Ensembl BioMart and MGI. For publication references refer to MGI. “Incomplete mouse data available” means not all orthologs have been knocked-out. ‡Genes that have been knocked-out are indicated in bold. *In instances where more than one mouse ortholog exists, the male infertile mouse ortholog is indicated in bold.
